# Analysis of risk factors of early intraventricular hemorrhage in very-low-birth-weight premature infants: a single center retrospective study

**DOI:** 10.1186/s12884-022-05245-2

**Published:** 2022-12-01

**Authors:** Ying Zhao, Wanxian Zhang, Xiuying Tian

**Affiliations:** Department of Neonatology, Tianjin Central Hospital of Gynecology and Obstetrics, Tianjin Key Laboratory of Human Development and Reproductive Regulation, Nankai University Maternity Hospital, No.156, Sanlu Road, Nankai district, Tianjin, 300052 China

**Keywords:** Retrospective analysis, Risk factors, IVH, VLBW, Premature infants

## Abstract

**Background:**

This study aimed to determine the risk factors of early intraventricular hemorrhage (IVH) in very-low-birth-weight (VLBW) premature infants in China to guide early interventions and improve the survival and quality of life of these infants.

**Methods:**

Data on 421 VLBW premature infants admitted to the neonatal intensive care unit of Tianjin Central Hospital of Gynecology Obstetrics between July 2017 and July 2019 were retrospectively evaluated. Data on head ultrasound results, maternal pregnancy complications, and perinatal conditions were reviewed to evaluate the association between maternal and neonatal factors and the development and severity of IVH.

**Results:**

Univariate analysis showed that the incidence of early IVH was significantly higher in neonates with early gestational age, delivered after spontaneous labor, low birth weight, 5-minute Apgar score ≤ 7, invasive mechanical ventilation, and early onset sepsis (χ^2^ = 11.087, 16.868, 4.779, 11.170, 6.655, and 6.260, respectively; *P* < 0.05), but it was significantly lower in the presence of gestational hypertension (χ^2^ = 4.373, *P* = 0.037). In addition, severe IVH was significantly associated with early gestational age, low birth weight, 5-minute Apgar score ≤ 7, and neonatal sepsis (χ^2^ = 11.599, 8.263, 11.172, and 7.749, respectively; *P* < 0.05). Logistic regression analysis showed that antenatal glucocorticoid use was associated with significantly reduced incidence of severe IVH (OR = 0.095, 95% CI = 0.012–0.739, *P* = 0.024).

**Conclusion:**

Appropriate mode of delivery may effectively reduce the incidence of IVH in VLBW premature infants. The antenatal glucocorticoid use may also protect against severe IVH. The focus on steroid prophylaxis, mode of delivery and prevention of perinatal asphyxia should be stressed in China.

## Background

Preterm infants are born at less than 37 weeks gestational age and low birth weight infants are born with a birth weight below 2.5 kg regardless of gestational age [1]. The low birth weight could be further stratified into low birth weight (1500–2500 g), very-low-birth-weight (VLBW, 1000–1499 g) and extremely low birth weight (ELBW, < 1000 g) [2]. Preterm birth accounts for the leading cause of mortality and morbidity in newborns and children under 5 years. With the constant development of medical technology, the survival rate of preterm infants has been increasing and the gestational age of viability in VLBW infants has been decreasing [3, 4]. The challenges in treating preterm infants with gestational age < 32 weeks have gradually evolved from increasing survival rates to improving quality of life.

Intraventricular hemorrhage (IVH) is one of the most common types of brain injury in preterm infants, especially in VLBW and ELBW infants [5, 6]. According to the Papile system, IVH is divided into grade I, defined as the presence of subependymal hemorrhages alone, grade II, defined as primary IVH without ventricular dilatation, grade III, defined as IVH with ventricular dilatation, and grade IV, defined as IVH with ventricular dilatation and brain parenchymal involvement [7]. Grade IV IVH is also specified as periventricular hemorrhage infarction or parenchymal hemorrhage in recent years. Grades I and II were considered mild IVH, while grades III and IV were considered severe IVH [8]. Recent studies have proposed that mild IVH is less likely to cause long-term neurological abnormalities, but severe IVH can negatively influence neurological development and result in death in preterm infants [9–11]. The known risk factors of IVH include gestational age, birth weight, asphyxia, hypoxia, infection, coagulation dysfunction, twin, placenta abnormalities such as placenta abruption, respiratory distress syndrome requiring surfactant treatment, intrauterine infection, disseminated intravascular coagulation, pneumothorax and mechanical ventilation [12–15]. Early prediction, diagnosis, interventions for IVH are essential for improving the survival and quality of life of premature infants.

Most IVH occurs within the first 72 hours after birth (early IVH) and progresses rapidly within 1 week [16]. Therefore, identification of risk factors associated with early IVH is important. Thus far, limited studies have evaluated the risk factors of early IVH by severity in VLBW or very premature infants in China, with majority of them being single-factor analyses.

In this study, we retrospectively analyzed the data on VLBW, very premature infants admitted to the neonatal intensive care unit (NICU) of our hospital. We aimed to determine the risk factors of IVH to guide clinicians in performing early intervention measures that can improve the survival and quality of life of VLBW preterm infants.

## Methods

### Patients

The clinical data of VLBW premature infants treated in the NICU of Tianjin Central Hospital of Gynecology Obstetrics between July 2017 and July 2019 were included in our study. The inclusion criteria were 1) admission to the NICU within 24 hours after birth, 2) gestational age < 32 weeks (very preterm), 3) birth weight < 1500 g (VLBW), and 4) head ultrasound (HUS) performed within 72 hours after birth. Premature infants with structural organ abnormalities, congenital metabolic diseases, and chromosomal abnormalities and those who died or were discharged within 72 hours after birth were excluded. This study was performed in accordance with the Declaration of Helsinki and approved by the Ethics Committee of our hospital (approval number, 2020KY048). Written informed consent to participate in this study was provided by the participants’ legal guardian/next of kin.

#### Delivery room management for premature infants

When a pregnant woman with GA < 32 weeks who is expected to have an unavoidable preterm birth is admitted to our center, the obstetrician consults the pediatrician, develops a treatment plan, and communicates with the relatives about the treatment plan. When childbirth begins, the pediatricians and anesthesiologists stand by in the delivery room and perform resuscitation and rescue for the newborn in need. After the child’s condition is relatively stable, it is transferred to NICU for further observation and treatment by the pediatricians. For infants with respiratory distress syndrome (RDS), pulmonary surfactant treatment under endotracheal- intubation (INSURE) is given. For infants present with dyspnea in the delivery room but do not require endotracheal intubation immediately, they are supported by noninvasive mechanical ventilation (MV) and are given INSURE in NICU if needed.

#### Ultrasound

HUS examinations were performed by a physician of the Department of Neonatology using color doppler ultrasound diagnostic system (Philips Epiq5, Philips Ultrasound, Inc., Washington, USA) within 72 hours after birth for all newborns. The infants were in the supine position and were scanned through the anterior fontanelle window for coronal and sagittal scanning. The diagnosis was established in communication between the department of ultrasound and the department of neonatology. If the neonatologist had doubts about the report, a consultation would be held among ultrasound doctor, neonatologist and the director of the ultrasound department. If more than one HUS was obtained within the first 72 hours, then the last HUS within 72 hours was used for this analysis. Severity of IVH was evaluated according to the Papile system.

### Data collection

Gestational age was defined based on results of the first prenatal fetal ultrasound, obstetric examination, maternal history evaluation, and neonatal gestational age assessment. In the case of a difference of > 2 weeks between the obstetric and neonatal gestational age assessments, the neonatal gestational age was considered. Gestational age of neonates age > 28 weeks was assessed using the simplified gestational age assessment table of China using the formula:

$$\textrm{Gestational}\ \textrm{age}\ \textrm{weeks}=\textrm{total}\ \textrm{score}+27$$

The total score (0–16 points) was assessed based on four aspects, including plantar skin creases (0–4 points), breast size (0–4 points), nail length (0–4 points) and skin texture (0–4 points) [17]. For the neonates aged < 28 weeks, gestational age was assessed using the New Ballard Score [18].

All relevant clinical data of infants and their mothers were retrospectively collected, including data on gestational age, sex, birth weight, mode of delivery, and the presence of pregnancy-related complications. Neonatal-related complications were also considered.

All study subjects were stratified according to selected factors. Gestational age was divided into ≤28 weeks, 28^+ 1^–30 weeks, and 30^+ 1^–32 weeks. Body weight was classified into ≤1000 g and > 1000 g. Mode of delivery included vaginal delivery, cesarean section, or vaginal breech delivery. The vaginal breech delivery means that the breech of fetus is exposed first and the midwife assists in the transposition of the fetus during vaginal delivery. Pregnancy-related complications included gestational hypertension (hypertension that develops in pregnancy after 20 weeks gestation and resolves within 12 weeks postpartum) [19], thyroid dysfunction [20], gestational diabetes mellitus [21], premature rupture of membranes (time from rupture of fetal membranes to delivery of the fetus ≥24 hours) [22], chorioamnionitis (serious non-specific infection by various pathogens mediated by placenta from the reproductive tract or blood) [23], and antenatal glucocorticoid use (6 mg intramuscular dexamethasone, Q12h, 4 doses). Neonatal-related complications identifiable within 7 hours of life included 5-minute Apgar score ≤ 7, early onset sepsis, hemorrhagic disease (a self-limited hemorrhagic disorder of the first day of life), patent ductus arteriosus (PDA, a persistent opening between the two major blood vessels leading from the heart, diagnosed by echocardiography within 72 h after birth), and the need for invasive or non-invasive mechanical ventilation.

### Statistical methods

Categorical data of the two groups are expressed as rate (%). Comparisons between the groups were performed using the χ^2^ test or Fisher’s exact test. All significant variables were included in multivariable logistic regression analysis, and the independent risk factors of IVH and its severity were determined accordingly. Three categories (vaginal delivery, vaginal breech delivery and caesarean section) in mode of delivery were merged into vaginal delivery and cesarean section for logistic regression. Omnibus test was used to test whether the explained variance in a set of data is significantly greater than the unexplained variance. The Hosmer-Lemeshow test was used to determine the goodness of fit of the logistic regression model. All statistical analyses were performed using IBM SPSS Statistics for Windows (version 23.0, IBM Corp, Armonk, NY, USA). *P* < 0.05 was considered statistically significant.

## Results

### Demographic characteristics of VLBW premature infants in IVH and non IVH groups

Of the 421 VLBW premature infants, 204 were males and 217 were females (male-to-female ratio: 0.94:1). There were 396 (94.1%) in-born infants and 25 (5.9%) out-born infants. The average gestational age was (29.20 ± 1.57) weeks. According to results of the 72-hour HUS, the subjects were divided into the IVH group (*n* = 86) and the non-IVH group (*n* = 335). No significant differences were found between the IVH and the non-IVH infants in sex, delivery site, maternal thyroid dysfunction, gestational diabetes mellitus, premature rupture of membranes, chorioamnionitis, primary hypotension of infants, antenatal use of glucocorticoids, respiratory distress syndrome, hemorrhagic disease, and PDA (all *P* > 0.05, Table 1). A total of 343 infants (81.5%) had RDS, including 74 infants in the IVH group and 269 infants in the non-IVH group. Surfactant application was performed by the INSURE procedure in all infants. Thirty-six infants (8.6%) had primary hypotension at birth, including 9 cases in IVH group and 27 cases in the non-IVH group. The blood pressure returned to normal level within 24 h after drug treatment.

**Table 1:** Demographic characteristics of Very Low Birth Weight premature infants in IVH and Non IVH groups

	IVH (*n* = 86)	Non IVH (*n* = 335)	χ2	*P*
Gestational age			11.087	0.004
≤ 28 weeks	30 (34.9%)	62 (18.5%)		
28^+ 1^–30 weeks	33 (38.4%)	173 (51.6%)		
30^+ 1^–32 weeks	23 (26.7%)	100 (29.9%)		
Sex			0.026	0.904
Male	41 (47.7%)	163 (48.7%)		
Female	45 (52.3%)	172 (51.3%)		
Birth weight			4.779	0.029
≤ 1000 g	22 (25.6%)	52 (15.5%)		
> 1000 g	64 (74.4%)	283 (84.5%)		
Delivery site			0.938	0.333
In-born	79 (91.9%)	317 (94.6%)		
Out-born	7 (8.1%)	18 (5.4%)		
Mode of delivery			16.868	< 0.001
Vaginal delivery	59 (68.6%)	150 (44.8%)		
Cesarean section	23 (26.7%)	172 (51.3%)		
Vaginal breech delivery	4 (4.7)	13 (3.9%)		
Gestational hypertension			4.373	0.037
Yes	4 (4.7%)	42 (12.5%)		
No	82 (95.3%)	293 (87.5%)		
Thyroid dysfunction			0.018	0.893
Yes	2 (2.3%)	7 (2.1%)		
No	84 (97.7%)	328 (97.9%)		
Gestational diabetes mellitus			0.052	0.819
Yes	16 (18.6%)	66 (19.7%)		
No	70 (81.4%)	269 (80.3%)		
Premature rupture of membranes			2.196	0.138
Yes	38 (44.2%)	119 (35.5%)		
No	48 (55.8%)	216 (64.5%)		
Chorioamnionitis			1.532	0.216
Yes	14 (16.3%)	75 (22.4%)		
No	72 (83.7%)	260 (77.6%)		
Primary hypotension			0.506	0.477
Yes	9 (10.5%)	27 (8.1%)		
No	77 (89.5%)	308 (91.9%)		
Antenatal use of glucocorticoids			0.051	0.822
Yes	33 (38.4%)	133 (39.7%)		
No	53 (61.6%)	202 (60.3%)		
Apgar score of 5 minutes after birth≤7			11.170	0.001
Yes	23 (26.7%)	41 (12.2%)		
No	63 (73.3%)	294 (87.8%)		
Respiratory distress syndrome			1.498	0.221
Yes	74 (86.0%)	269 (80.3%)		
No	12 (14.0%)	66 (19.7%)		
Early onset sepsis			6.260	0.012
Yes	20 (23.3%)	42 (12.5%)		
No	66 (76.7%)	293 (87.5%)		
Hemorrhagic disease			0.078	0.780
Yes	8 (9.3%)	28 (8.4%)		
No	78 (90.7%)	307 (91.6%)		
PDA			1.600	0.206
Yes	33 (38.4%)	154 (46.0%)		
No	53 (61.6%)	181 (54.0%)		
Mechanical ventilation			6.655	0.036
IMV	7 (8.1%)	9 (2.7%)		
NIMV	71 (82.6%)	278 (83.0%)		
Non MV	8 (9.3%)	48 (14.3%)		

*IVH* Intraventricular Hemorrhage, *PDA* Patent ductus arteriosus, *IMV* Invasive mechanical ventilation, *NIMV* Non-invasive mechanical ventilation

The incidences of IVH in the ≤28, 28^+ 1^–30, and 30^+ 1^–32 weeks groups were 32.6% (30/92), 16.0% (33/206), and 18.7% (23/123), respectively, with significant difference observed among the groups (χ^2^ = 11.087, *P* = 0.004). Other significant risk factors for any IVH in VLBW premature infants included the mode of delivery (*P* < 0.001), birth weight (*P* = 0.029), 5-minute Apgar score (*P* = 0.001), neonatal sepsis (*P* = 0.012), mechanical ventilation (*P* = 0.036), and gestational hypertension (*P* = 0.037). All details are shown in Table 1.

### Risk factors for any IVH in VLBW premature infants

The significant risk factors in univariate analysis, including gestational age, birth weight, mode of delivery, gestational hypertension, 5-minute Apgar score, neonatal sepsis, and mechanical ventilation were included in the multivariable logistic regression analysis. Mode of delivery (OR = 2.727, 95% CI = 1.522–4.885, *P* = 0.001] and 5-minute Apgar score ≤ 7 (OR = 2.273, 95%CI = 1.163–4.442, *P* = 0.016] were found to be independent risk factors for IVH, as shown in Table 2. The significance value of Omnibus test indicated the current model outperforms the null model (χ^2^ = 37.634, *P* < 0.001). Hosmer & Lemeshow test (χ^2^ = 6.710, *P* = 0.568) indicated the model fits the data well.

**Table 2:** Analysis of influencing factors of any intraventricular hemorrhage in very low birth weight infants

Variables	β	SE	Wald *χ*^2^	OR (95% CI)	*P* value
Gestational age			2.992		0.224
≤ 28 weeks	−0.556	0.322	2.976	0.574 (0.305–1.079)	0.084
28^+ 1^–30 weeks	−0.370	0.425	0.758	0.691 (0.300–1.589)	0.384
Body weight	−0.505	0.349	2.099	0.603 (0.305–1.195)	0.147
Mode of delivery	1.003	0.297	11.378	2.727 (1.522–4.885)	0.001
Gestational hypertension	− 0.666	0.563	1.398	0.514 (0.170–1.550)	0.237
5-minute Apgar score after birth ≤7	0.821	0.342	5.768	2.273 (1.163–4.442)	0.016
Early onset sepsisPremature sepsis	0.550	0.325	2.859	1.734 (0.916–3.281)	0.091
Mechanical ventilation	0.616	0.573	1.157	1.852 (0.603–5.693)	0.282

*SE* Standard error, *OR* Odds ratio, *CI* Confidence interval. Three categories in mode of delivery were collapsed into vaginal delivery and cesarean section for logistic regression

### Factors associated with severe IVH in VLBW premature infants

To explore the association of risk factors with severe IVH, the 421 subjects were further divided into severe (IVH grades III–IV, *n* = 19) and mild IVH groups (IVH grades I–II and non-IVH, *n* = 402). The male-to-female ratios of the severe and mild IVH groups were 0.90:1 and 0.94:1, respectively. Based on univariate analysis, the incidence rate of severe IVH was 12.2% (10/92), 3.4% (7/206) and 1.6% (2/123) in premature infants with gestational age of ≤28 weeks, 28^+ 1^–30 weeks, and 30^+ 1^–32 weeks, respectively. Subgroup analysis showed that the incidence rate of severe IVH was significantly higher in the ≤28 weeks group than that in the other two groups (*P* < 0.05), with no significant difference observed between the other two groups (*P* > 0.05).

The incidence of severe IVH in premature infants with birth weight ≤ 1000 g was higher than that that in infants with birth weight > 1000 g (10.8% vs. 3.2%, χ^2^ = 4.779, *P* = 0.004). The incidence rate of severe IVH in premature infants with 5-minute Apgar score ≤ 7 and those with early onset sepsis was significantly higher than that in premature infants with 5-minute Apgar score > 7 (12.5% vs. 3.1%, χ^2^ = 11.172, *P* = 0.001) and those without sepsis (11.3% vs. 3.3%, χ^2^ = 7.749, *P* = 0.005). Antenatal glucocorticoid use was associated with significantly lower rates of severe IVH than non-use (0.6% vs. 7.1%, χ^2^ = 9.726, *P* = 0.002). However, factors such as sex, delivery mode, thyroid dysfunction during pregnancy, gestational diabetes mellitus, chorioamnionitis, premature rupture of membranes, neonatal hemorrhagic diseases, postnatal infection, PDA, and invasive mechanical ventilation had no significant influence on the incidence rate of severe IVH in VLBW premature infants.

Factors those were significantly associated with the severe IVH in univariate analysis, such as gestational age, birth weight, 5-minute Apgar score, early onset sepsis, and antenatal glucocorticoid use, were included in multivariable logistic regression analysis (Table 3). Antenatal glucocorticoid use (OR = 0.095, 95% CI = 0.012–0.739, *P* = 0.024) was found to be an independent risk factor for severe IVH in VLBW premature infants. The significance value of Omnibus test indicated the current model outperforms the null model (χ^2^ = 32.191, *P* < 0.001). Hosmer & Lemeshow test (χ^2^ = 3.617, *P* = 0.890) indicated the model fits the data well.

**Table 3:** Influencing factors of severe intraventricular hemorrhage in low birth weight infants

Variables	β	SE	Wald *χ*^2^	OR (95% CI)	*P* value
Gestational age			1.167		0.558
≤ 28 weeks	0.671	0.834	0.649	1.957 (0.382–10.030)	0.421
28^+ 1^–30 weeks	1.052	0.974	1.166	2.863 (0.424–19.325)	0.280
Body weight	−0.741	0.585	1.600	0.477 (0.151–1.502	0.206
Mode of delivery	0.173	0.583	0.088	1.189 (0.379–3.730)	0.767
Apgar score of 5 minutes after birth ≤7	0.875	0.581	2.265	2.399 (0.768–7.499)	0.132
Early onset sepsisPremature sepsis	0.918	0.545	2.838	2.505 (0.861–7.290)	0.092
Mechanical ventilation	0.598	0.945	0.400	1.819 (0.285–11.603)	0.527
Antenatal use of glucocorticoids	−2.349	1.044	5.061	0.095 (0.012–0.739)	0.024
Gestational hypertension	−17.148	5384.8	< 0.001	0 (0 - -)	0.997

*SE* Standard error, *OR* Odds ratio, *CI* Confidence interval

## Discussion

This study included 421 VLBW, very premature infants and assessed the presence and severity of IVH using HUS within 72 hours after birth. Multivariable logistic regression analyses suggested that 5-minute Apgar score ≤ 7 [OR = 2.273, 95%CI = 1.163–4.442, *P* = 0.016] and mode of delivery (OR = 2.727, 95% CI = 1.522–4.885, *P* = 0.001] were the independent risk factors of any severity of IVH in VLBW preterm infants and that antenatal glucocorticoid use significantly reduced the risk of severe IVH (OR = 0.095, 95%CI =0.012–0.739, *P* = 0.024).

Low Apgar scores are well known to be a risk factor of IVH [24–27]. The results of this study were in accordance with those of other studies. In preterm infants, the germinal matrix of the periventricular subependymal zone and the cerebellar submeningeal granular layer are not fully mature and the surrounding layer of thin-walled capillaries in the germinal matrix lacks supporting tissues [28]. In the case of hypoxia, spontaneous perforation and pressure rupture of the immature vascular walls lead to hemorrhage. Moreover, immaturity of the cerebrovascular autoregulatory systems of premature infants further increase the risk of cerebral ischemia caused by systemic hypotension during asphyxia and hypoxia, resulting in a greater risk of damage to the blood vessels of the germinal matrix [28].

There is currently no international consensus on the effect of delivery mode on IVH in children weighing less than 1500 g. Riskin et al. demonstrated that the odds for severe IVH were not influenced by the mode of delivery in vertex-presenting singleton VLBW infants [29]. In contrast, vaginal delivery was associated with an increased incidence of IVH (28.2–23.5% vs. 11.8%) in our patients, consistent with the findings reported by Gamaleldin et al. [30]. Similarly, Humberg et al. [31] demonstrated that vaginal delivery of preterm infants at a gestational age of < 30 weeks significantly increased the risk of IVH (OR = 1.725, 95% CI = 1.325–2.202, *P* < 0.001). This may be attributable to the dramatic changes in vascular pressure involved during vaginal deliveries, in which capillary pressure increases during vaginal extrusion of the head and immediately decreases thereafter. Under such circumstances, capillary rupture tends to occur in the immature germinal matrix. Therefore, cesarean section might reduce the incidence of IVH in preterm infants of early gestational age. In the present study, vaginal delivery was significantly associated with IVH, but not significantly associated with severe IVH. During recent years, China implemented the ‘two-child policy’ and ‘three-child policy’ gradually, which means that a couple can have two or three children. For the women delivering premature infants, most of them prefer vaginal delivery in order to preserve the possibility of having a subsequent child by vaginal delivery rather than repeat cesarean section. This study shows that vaginal delivery increases the risk of IVH. In addition, the number of women of advanced maternal age in China increased. These women were more likely to be overweight, having gestational diabetes and gestational hypertension. They had worse perinatal outcomes such as preterm delivery, low birthweight babies, higher rates of Neonatal Intensive Care Unit admission and worse Apgar scores [32]. Therefore, this study might help improve the survival and quality of life in preterm infants with early gestational age in the centers such as ours in China. Since the number of severe IVH is relatively small, further studies with larger samples are warranted to confirm this conclusion.

For preterm infants with RDS, exogenous surfactant replacement therapy is needed [33]. The most popular method is the INSURE technique, which allows surfactant being applied without ongoing MV and leads to a reduced rate of bronchopulmonary dysplasia [34]. In this study, 343 preterm infants had RDS, including 74 infants in the IVH group and 269 infants in the non-IVH group. All these infants were treated with INSURE technique. However, some infants still need invasive MV, which has been found to be one of the factors leading to IVH in VLBW premature infants [35–37]. Although invasive MV was found to be associated with an increased rate of IVH in this study, it was not shown to be an independent risk factor in multivariate logistic analysis. This could have been due to the small sample size included in our study. In recent years, less invasive surfactant administration (LISA), in which exogenous surfactant was applied by a thin catheter on nasal continuous positive airway pressure was associated with higher survival rates and lead to fewer complications such as severe IVH [38, 39]. However, the infants in this study were treated with INSURE, not LISA method because the later was introduced to our center in 2020.

This study shows that antenatal glucocorticoid use (6 mg intramuscular dexamethasone, Q12h, 4 doses) can remarkably reduce the risk of severe IVH in VLBW preterm infants (OR = 0.083, *P* = 0.018). Glucocorticoids, as drugs to promote fetal lung maturation, have been widely used for the prevention of neonatal respiratory distress syndrome. In recent years, many studies have found that antenatal glucocorticoid use can reduce the incidence of IVH in premature infants [10, 40–42]. This may be due to the role of glucocorticoids in facilitating fetal vasoconstriction and alleviating fetal vasodilation, which would have been a cause of IVH during the hypercapnic state [40]. Therefore, prenatal glucocorticoid use might improve the survival and quality of life in preterm infants.

We attempted to analyze the risk factors of IVH in our institution to identify the gaps of practice to improve the care of VLWB infants. However, there are some limitations in this study. First, this is a single center retrospective study with a small number of patients. Especially, the number of severe IVH is only 19. Second, some bias, such as confounding bias and temporal bias might exist in the results of our analysis because of the nature of retrospective study. Therefore, multicenter studies with larger sample sizes are warranted to obtain more accurate results.

Through the analysis of 421 preterm infants born at a gestational age of < 32 weeks, we showed that mode of delivery and 5-minute Apgar score ≤ 7 are the risk factors of IVH, while antenatal glucocorticoid use plays a protective role against severe IVH. Therefore, the focus on steroid prophylaxis, mode of delivery and prevention of perinatal asphyxia should be stressed in China. More clinical attention should be placed on these factors to help improve the survival and quality of life in preterm infants with early gestational age.

## Data Availability

The datasets generated and/or analysed during the current study are not publicly available due to the participants of this study did not agree to their data being shared publicly, but are available from the corresponding author on reasonable request.

## Abbreviations

IVH: Intraventricular hemorrhage
VLBW: Very low birth weight
NICU: Neonatal intensive care unit
OR: Odds ratio
CI: Confidence interval
PDA: Patent ductus arteriosus
